# Applicability of Demirjian’s method for dental age estimation in a group of Egyptian children

**DOI:** 10.1038/s41405-019-0015-y

**Published:** 2019-03-21

**Authors:** Amro M. Moness Ali, Wael H. Ahmed, Nagwa M. Khattab

**Affiliations:** Faculty of Dentistry, Pediatric and Community Dentistry Departmenty, Minia University, Minia, 61111 Egypt

## Abstract

**Aims:**

The aims of this study were to evaluate the applicability of Demirjian’s method for dental age assessment in a group of Egyptian children in Minia city and to develop an age predictive equation suitable for the studied group.

**Subjects and methods:**

In this retrospective, blind, cross-sectional study, 160 dental panoramic radiographs (DPTs) were selected from 420 DPTs from healthy children aged between three and 10 years old from the archived medical files of patients attending Minia University Dental Hospital (MUDH) and evaluated to estimate dental ages.

**Results:**

Age was overestimated for almost all of the studied subjects with an accuracy range from 0.18 to 1.19 years for males and from 0.08 to 0.87 years for females, with the exception of two age subgroups (9–10-year-old males and 10–11-year-old females, for which the mean difference values were −0.06 and −0.008 years, respectively). A Logistic regression was used to generate a suggested formula for dental age estimation.

**Conclusions:**

Demirjian’s method may be unsuitable for Egyptian children living in Minia city. Development of a predication equation and the introduction of adaptable conversion tables to transform the maturity score into a dental age for Egyptian children may be suitable alternatives. The validity of the newly developed prediction equation must be tested among all Egyptian children.

## Introduction

The ages of juveniles and adolescents can be estimated using skeletal and dental anthropological methods. Dental development is a helpful indicator of maturation due to its high reliability, low coefficient of variation, and resistance to environmental effects.^1^

Typically, dental age (DA) estimations in children are based on clinical examinations that include recordings of tooth eruption or observations of the tooth formation stages using radiographs.^2,3^ The radiographic method is much more accurate than the clinical method, because tooth emergence is a short period that is determined by the time of tooth appearance in the mouth and can be altered by local factors, such as a lack of space, and systemic factors, such as the nutritional status.^4^

Several dental development determination methods using radiographs have been described.^5–7^ Most of these methods are based on comparisons between the radiographic developmental stages of a tooth and standard charts compiled from a large population in a well-defined geographic region.^7^

One of the most widely applied methods is the Demirjian system, which was first described in 1973 and was based on a sample of French-Canadian children. Demirjian’s method is theoretically based on eight developmental stages ranging from crown and root formation to apex closure of the seven left permanent mandibular teeth. The score of each stage is allocated, and the sum of the scores provides an evaluation of the subject’s dental maturity. The dental maturity score (DMS) can be converted into the DA using available tables. Then, the percentile curves from the original study are allocated, and the sum of the scores provides an evaluation of the subject’s dental maturity. A difference between the dental and chronological age (CA) of a subject indicates an advancement or delay in dental maturity.^4^

Numerous researchers have applied this method to groups of children in various areas worldwide, and significant differences between most groups and the reference group have been interpreted as either true population differences or secular trends. Many authors have used these differences to justify the need for a population-specific DMS.^8–11^ Demirjian’s method applicability was reported by many authors, revealing a considerable matter of debate about its applicability to all races and different population.^1,2,12–16^

## Aims of the study

The aims of this study were as follows (Fig. S1):

- To evaluate the applicability of Demirjian’s method for DA assessment in a group of Egyptian children in Minia city and
- To develop an age predictive equation suitable for the studied group.

## Subjects and methods

This study is a blind retrospective cross-sectional study.

### Sampling

Dental panoramic radiographs (DPTs) from healthy children aged between 3 and 10 years old were chosen from the archived medical files of patients attending Minia University Dental Hospital (MUDH) between February and December 2017. The DPTs were previously taken for diagnostic purposes and were reused in this study.

### The inclusion criteria were as follows:

- The CAs of the participants were between 3 and 10 years old;
- The child’s parent(s) or caregiver contact information was available to obtain a record of the child’s medical history; and
- Both the date of birth (DOB) and the date of the radiograph (DOR) were available.

### The exclusion criteria were subjects with

- Systemic diseases or genetic disorders that would affect skeletal and dental growth;
- Localized oral pathology, anomalies or impacted teeth that would affect dental growth;
- Severe malocclusion;
- A history of current or previous orthodontic treatment; and
- a DPT of poor quality in which one or more targeted teeth could not be scored.

### Study groups

The selected DPTs were divided into two main groups according to biological sex [Group A (males) and Group B (females)]. Further subgrouping was performed according to the child’s age, with the main groups divided into seven age levels (eight subgroups) at yearly intervals (exclusive type class interval) with at least five participants per age group.

### Data collection

DPT and personal information related to the CA of each subject, such as the DOB and DOR, were collected from the existing records. Each DPT was assigned a code, scanned at a resolution of 300 dpi in gray-scale format, and stored as a JPEG image with dimensions of 2440 × 1280 pixels (Epson scanner 1000XL, Epson Inc., USA). The CAs of the participants were calculated by subtracting the DOB from the DOR and were recorded as years with two decimal places.^17^

### Scoring of the radiographs

- All of the DPTs were scored independently and randomly (using electronically generated random numbers) by one of the authors, who was blinded to the CA and sex of each subject.
- The digitized DPT was viewed on a widescreen monitor with Microsoft Office Picture Manager 2010 (Microsoft Corp., USA); when required, the DPT was magnified up to two times for identification of the dental development stages.^17^
- The DA was calculated using Demirjian’s method. All of the teeth in the lower left jaw (with the exception of the third molar) were assessed.^18^ The DA was calculated according to the tables proposed by Demirjian et al.^4^ When a tooth on one side was missing or difficult to read, the contralateral tooth was assessed. A Microsoft Excel 2010 (Microsoft Corp., USA) database was used for data entry.

### Reproducibility

Twenty percent of the DPTs were randomly selected using electronically generated random numbers, and the tooth developmental stages were re-evaluated two weeks later (retest) to test the inter-examiner and intra-examiner reliability. Then, the intra- and inter-examiner agreement was calculated.

### Sample size and power analysis

The required sample size was estimated using OpenEpi, Version 3, open source calculator—SSPropor based on the following formula^19^

$${\mathrm{Sample}}\,{\mathrm{size}}\,n = \left[ {\mathrm{DEFF}}^{\ast} {\mathrm{Np}}\left( {{\mathrm{1 - p}}} \right) \right]/\left[ \left( {\mathrm{d2/Z21 - \alpha /2}}^{\ast} \left( {{\mathrm{N - 1}}} \right) \right. \right. \\ \left. + \, {\mathrm{p}}^{\ast} \left( {{\mathrm{1 - p}}} \right)\right]$$

In which; population size (for finite population correction factor) (N), hypothesized % frequency of outcome factor in the population (p) was 50% ±5, confidence limits as % of 100 (absolute ± %) (d) was 5%, design effect for cluster surveys (DEFF) was 1, value of Z obtained from statistical tables corresponding to 95% confidence interval was 1.96 and the degree of precision (α) was 0.05.

### Statistical testing

All data were collected, tabulated, and statistically analysed using SPSS version 20 (Armonk, NY: IBM Corp. USA). Quantitative data are presented as the range, mean, and standard deviation (SD), and qualitative data are presented as the number (*n*) and percentage (%). The statistical analyses were performed using an independent samples *t*-test for analysis of quantitative data and a scatter plot with a regression line for the association analyses. For all tests, probability (*p*) was categorized as follows:

- Non-significant if ≥0.05;
- Significant if <0.05;
- Highly significant if <0.01; and
- Very highly significant if <0.001.

Cohen’s kappa test with a *p*-value < 0.05 indicating significance was used to test the inter-examiner and intra-examiner reliability.

### Ethical regulation

- Ethical approval was granted by the Ethics Committee of the Faculty of Dentistry of Minia University, Egypt, on 27/2/2018 and was registered under number 204. The procedures followed were in accordance with the Declaration of Helsinki of 1975 as revised in 2000.
- Prior to collecting DPTs, the child’s parent(s) or caregiver(s) was/were asked for written permission approving the use of the child’s radiographic and personal data.

## Results

Only 169 out of a total of 420 DPTs satisfied the selection criteria of the current study; moreover, after interpretation of the selected DPTs, nine cases were excluded because their radiographic interpretations revealed DAs of less than 2.5 years, which was outside the range of our study. Therefore, 160 DPTs were included in the study, as shown in Table 1.

**Table 1:** Distribution of the studied children by age and biological sex

	Males		Females	
Age groups	Frequency	Percent (%)	Frequency	Percent (%)
3 to <4	10	13.5	8	9.3
4 to <	10	13.5	9	10.5
5 to <6	11	14.9	15	17.4
6 to <7	5	6.8	10	11.6
7 to <8	10	13.5	10	11.6
8 to <9	10	13.5	10	11.6
9 to <10	8	10.8	12	14.0
10 to <11	10	13.5	12	14.0
Total	74	100.0	86	100.0
*P*-value	0.114		0.119	

### Reproducibility

Inter-examiner reliability was assessed by correlating the data obtained from the test and retest processes. The linear Pearson’s correlation between the test and retest resulted in *P*-values less than 0.001, indicating that the ratings were significantly correlated. The percentage of intra-observer agreement for 32 DPTs was 89.25%, with eighteen one stage ahead and fourteen one stage behind.

### Applicability of Demirjian’s method

The application of Demirjian’s method for DA estimation among the studied group revealed statistically significant differences among the 3–4, 5–6, 7–8, 9–10, and 10–11 age groups in males and the 3–4, 5–6, and 6–7 age groups in females. In the 4–5 and 8–9 age groups, overestimation was noted in both sexes, whereas overestimation was observed in only one gender in the other groups (Table 2).

**Table 2:** Comparisons between the estimated dental ages (EDA) and chronological ages (CA) among the studied children using an independent samples *t*-test

Age group	Gender	Mean ±SD		Mean difference (EDA-CA)	*P*-value
		CA	EDA		
3–4	Male	3.21 ± 0.35	4.40 ± 1.04	1.19	**0.006** ^a^
	Female	3.67 ± 0.3	4.55 ± 0.79	0.87	**0.017** ^b^
4–5	Male	4.39 ± 0.39	4.64 ± 0.81	0.25	**0.393** ^d^
	Female	4.08 ± 0.16	4.68 ± 0.78	0.60	**0.052** ^d^
5–6	Male	5.08 ± 0.27	5.99 ± 0.66	0.90	**0.001** ^a^
	Female	5.32 ± 0.29	5.92 ± 0.83	0.60	**0.018** ^b^
6–7	Male	6 ± 0	6.1 ± 1.1	0.18	**0.725** ^d^
	Female	6.42 ± 0.27	6.86 ± 0.45	0.44	**0.018** ^b^
7–8	Male	7.28 ± 0.32	7.86 ± 0.27	0.58	**<0.001** ^c^
	Female	7.34 ± 0.31	7.57 ± 0.30	0.23	**0.118** ^d^
8–9	Male	8.35 ± 0.28	8.6 ± 0.38	0.25	**0.116** ^d^
	Female	8.28 ± 0.33	8.36 ± 0.52	0.08	**0.691** ^d^
9–10	Male	9 ± 0	8.4 ± 0.39	−0.60	**0.003** ^a^
	Female	9.20 ± 0.23	9.14 ± 0.47	−0.06	**0.671** ^d^
10–11	Male	10.21 ± 0.25	10.7 ± 0.35	0.57	**0.001** ^a^
	Female	10.42 ± 0.32	10.41 ± 0.50	−0.008	**0.962** ^d^
Total	Male	6.37 ± 2.20	6.85 ± 2.05	0.466	**0.192** ^d^
	Females	7.11 ± 2.21	7.44 ± 1.96	0. 325	**0.313** ^d^

^a^Highly significant difference, *P*-value <0.01

^b^Significant difference, *P*-value <0.05

^c^Very highly significant difference, *P*-value <0.001

^d^Non-significant difference, *P*-value ≥0.05

Because estimated dental age (EDA) could not be applied to all age groups within our sample, a prediction equation was formulated.

### Development of a prediction equation

#### Estimation of the differences between EDAs and CAs

**Fig. 1:**
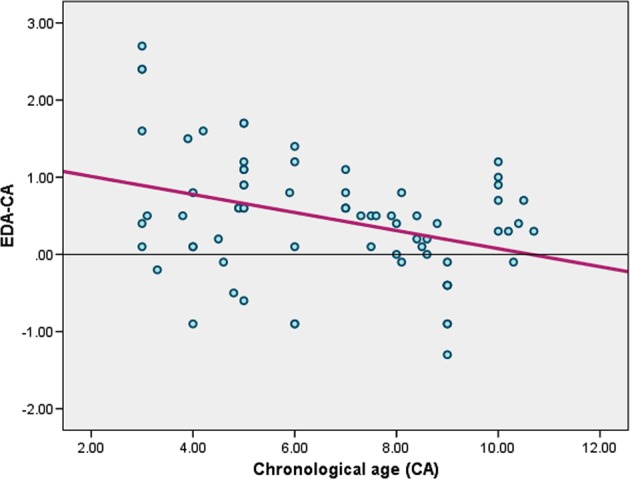
Scatter plot showing the differences between the estimated dental ages and the chronological ages (EDA-CA) plotted against the chronological ages (CAs) with a regression line for the male group

**Fig. 2:**
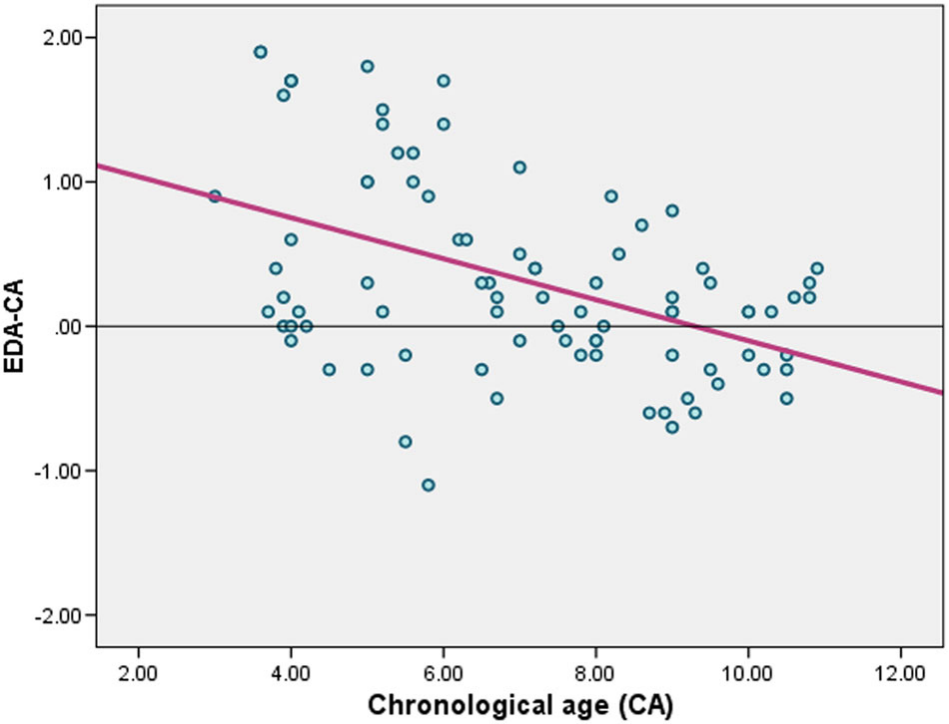
Scatter plot showing the differences between the estimated dental ages and chronological ages (EDA − CA) against the chronological ages (CAs) with a regression line for the female group

The differences between the EDAs and the CAs (EDA − CA) were plotted against the CAs. Each bn represents one child. The smallest values (~0) represent children whose EDAs were close to their CAs. For the male group, values above zero refer to children whose EDAs are overestimated (maximum of 2.7 years), and values below zero refer to children whose DAs are underestimated (maximum 1.3 years). The EDA was found to overestimate age with a mean difference of 0.4662 ( ± 0.78675) years from the CA among the studied males (Fig. 1). Similar values were observed for the female group, whose EDAs were also overestimated (maximum of 1.9 years); similarly, values below zero refer to children whose DAs were underestimated (maximum of 1.1 years). Among the females, the EDA was found to overestimate age with a mean difference of 0.3256 ( ± 0.6920) years from the CA (Fig. 2).

#### Correlation between the DMS and CA

**Fig. 3:**
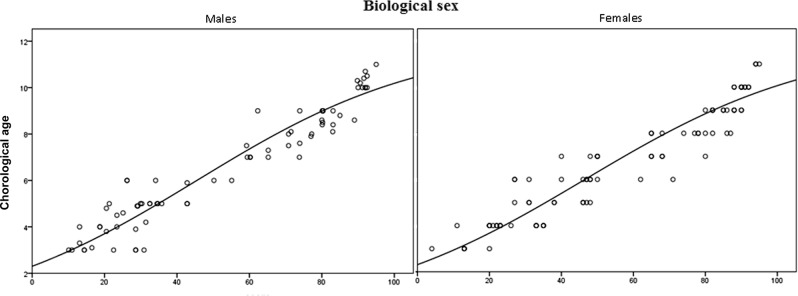
Regressions of the mean chronological age versus the dental maturity score for males and females

A Logistic regression analysis was performed to investigate the relationship between the DMS and the CA. The scatter plot graph showed a strong positive relationship between the two measures, which was confirmed by Spearman’s correlation coefficients of 0.947 and 0.935 for the males and females, respectively. Logistic regression showed a significant relationship between the DMS and the CA (*p* < 0.001) for both sexes. The slope coefficients for the DMS were 0.969 and 0.970 for males and females, respectively, indicating that the CA increased by 0.969 and 0.970 years for each extra unit of DMS for males and females, respectively. The *R*^2^ values were 0.897 and 0.874, indicating that 89.7 and 87.4% of the variation in CA for males and females, respectively, could be explained by the logistic model containing only the DMS (Fig. 3).

Thus, the suggested formulas for age prediction according to the interpreted data are as follows:

The equation for males is:

$${\mathrm{CA = 1/}}\left( {{\mathrm{0}}{\mathrm{.083 + 0}}{\mathrm{.351 \times 0}}{\mathrm{.969}}^{{\mathrm{DMS}}}} \right)$$

The equation for females is:

$${\mathrm{CA = 1/}}\left( {{\mathrm{0}}{\mathrm{.083 + 0}}{\mathrm{.350 \times 0}}{\mathrm{.970}}^{{\mathrm{DMS}}}} \right)$$

## Discussion

Age assessment is frequently required for medical odonatological purposes to predict the optimal time for treatment and especially for forensic purposes. Therefore, the estimated age should be as accurate as possible.^7,20^

DA estimation is commonly used worldwide and is thought to correlate with CA better than other maturity indicators of a child’s development.^21^ Several methods have been introduced to estimate DA depending on either calcification (tooth development)^7,20,22,23^ or eruption patterns.^24^ Relying on eruption dates when attempting to assess DA is complicated by the fact that tooth emergence may be significantly affected by local exogenous factors, such as infection, obstruction, crowding, and premature extraction of the deciduous predecessor or adjacent permanent teeth.^25^ These mishaps can be avoided by interpreting radiographic data representing the tooth development stages.

One of the most commonly used radiographic methods is the method reported by Demirjian et al., which established a standard based on a large sample that included 1446 males and 1482 females of French-Canadian origin.^4^ Although observer agreement is usually reported when using Demirjian’s method, there is an evident tendency towards overestimation of a subject’s age,^7^ which may be a result of ethnic differences between populations^26^ and a positive secular trend over the last 50 years.^27^ The debate regarding the applicability of Demirjian’s method to all races and populations.^1,2,12–16^ encouraged the authors to assess the applicability of Demirjian’s method and to develop new prediction equations, if needed.

In the current study, the inter- and intra-observer agreement was satisfactory, denoting the reliability of radiographic interpretation. In addition, statistical testing using linear regression was conducted to modify the maturity scores generated using Demirjian’s method. Logistic regression analysis may be a suitable method when it is needed to assign a subject with a specific age.^10,27^

Intraoral radiographs are usually predisposed to image distortion; therefore, archived DPTs were used because they were not only accessible but also enabled visualization of all of the teeth together, which was the recommended method reported by Demirjian et al.^4,21^

Although our sample size appears small compared to those of similar studies, a small sample size is not considered a limitation in forensic scientific research.^28^ Moreover, our sample size was larger or relatively equal to those of other studies. These studies included a cross-sectional study that compared EDAs with CAs in 162 Somali and white Caucasian children residing in Sheffield. The outcomes of that study highlighted the need for population-specific dental development standards for accurate assessment of DA.^29^ Likewise, Prabhakar et al. tested the applicability of Demirjian’s method among 151 Indian children living in Davangere. They found that the Davangere children were dentally more advanced and that Demirjian’s method was not applicable to their study group.^30^ Other studies with larger sample sizes than ours, including those that surveyed older age groups, recommended creation of an adaptive tool to avoid the overestimation observed using Demirjian’s method.^1,3,10,31,32^

Our results revealed an overestimation by 0.466 years in the male group, which was similar to the results obtained using similar age groups of Serbian (0.45 years),^9^ Dutch (0.4 years),^33^ and French (0.47 years) males.^34^ In addition, the currents results are in accordance with those of studies reported for Iranian (0.34 years)^35^ and southern Turkish (0.52 years) children,^15^ albeit to a lower intensity. In the female group, a mean difference of 0.325 years was calculated between the EDAs and CAs. Similar findings were reported among females living in Tanta, Egypt (0.294 years)^32^ and Norway (0.3 years).^36^ This coherency is most likely attributed to the fact that Egyptians, similar to many European populations, are all European-ancestry populations and share more or less the same geographical characteristics.^37^

The reverse observations were reported for South Indians (3.04 years in males and 2.82 years in females),^26^ Saudis living in Rayed (0.3 years for males and 0.4 years for females),^31^ Kuwaitis (0.71 years for males and 0.67 years for females),^38^ and Tunisians (from 0.3 to 1.32 year for males and from 0.26 to 1.37 year for females).^10^ The differences in age estimation between our study and those of other studies may be related to differences in the sample size, age groups, and studied populations. Other factors, such as socioeconomic status, nutrition, and dietary habits, may also affect the outcomes.^10^

The results of the current study revealed that dental maturation was more advanced in the examined males than in the studied females (mean differences between EDAs and CAs of 0.466 and 0.325 years for males and females, respectively). In addition to the absence of a significant difference between the male and female groups, the sexual dimorphism of the acceleration of dental maturation estimated by Demirjian’s method differed in numerous studies. Some researchers have reported acceleration of the EDA in females compared to that in males.^10,32,39^ However, the EDA among males could be in advance of that in females, as reported by Duangto et al.,^40^ who examined a Thai population and found mean differences of 0.11 and 0.10 years for males and females, respectively. In addition, Gungor et al.^15^ evaluated the applicability of Demirjian’s method for an elderly southern Turkish population and reported that the mean differences between the chronological and DAs ranged from 0.04 to 0.85 years and from 0.02 to 0.79 years in males and females, respectively. Moreover, a cross-sectional study among the Malay population clearly stated that Demirjian’s method overestimated the age by mean differences of 0.75 and 0.61 years among males and females, respectively.^21^

Although the current results and other reports have suggested that Demirjian’s method can be unsuitable as a forensic age estimation tool,^7,23^ Demirjian’s method is still a recommended method to assess individual dental maturity.^41^ Notably, no method has the ability to accurately determine the exact CA, because differences between the EDA and CA appear not only due to the accuracy of the applied method but also due to other factors, such as the examiners’ skills and experience, the studied sample size and distribution, developmental and environmental variability between the studied subjects themselves, and the methods used to analyze and interpret the obtained results.^42^

Our study showed the absence of a specific trend in addition to significant differences between some of the studied age groups. Thus, we found that Demirjian’s original standards did not accurately estimate the CA in our studied sample and that the EDA generally overestimated the CA upon application of Demirjian’s method in many populations. The authors strongly believe that each population requires its own adaptive dental maturity score. This concept of developing a specific prediction equation for each population is becoming more strongly supported.^16^

A limitation of the current study was that because our sample only represents Minia city, it may not represent the general Egyptian population. Therefore, the developed prediction equation requires modifications prior to application to the whole Egyptian population. Moreover, other limitations were the clinical nature of the sample, the age range of the children, three to 10 years, which includes very young individuals showing the earlier stages of permanent tooth development that are typically not part of such studies because of the ethical issues associated with radiography of children under five years; small cohort sizes; and the cross-sectional nature of the data, which is always a limitation when examining growth patterns.

## Conclusions

Within the limitation of this study, the authors conclude the following:

- Demirjian’s method and standards could be unsuitable for Egyptian children living in Minia city.
- Development of a predication equation and introduction of adaptable conversion tables for transformation of the maturity score into DA for Egyptian children could be a suitable alternative.
- The validity of the newly developed prediction equation must be tested among all Egyptian children.

## Supplementary information

Figure S1
