# Supplementary material for: Applicability of Demirjian’s method for dental age estimation in a group of Egyptian children
Source: BDJ Open. 2019 Mar 21;5:2. doi: 10.1038/s41405-019-0015-y (PMC6430778; doi:10.1038/s41405-019-0015-y)
Supplement: Supplementary file 1 — Figure S1 [file 41405_2019_15_MOESM1_ESM.pdf]

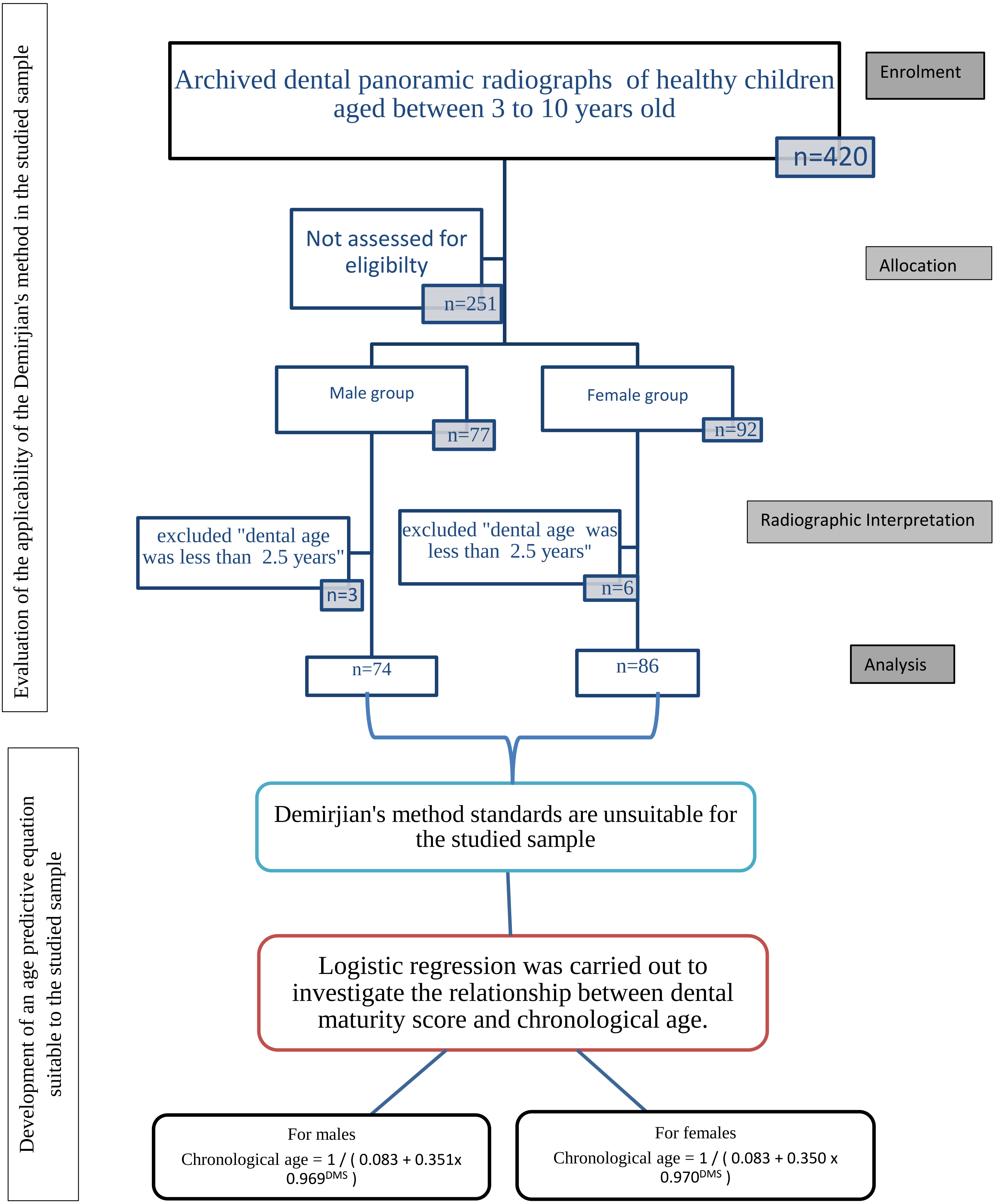

DMS=Dental Maturity score

Figure S1. Graphical abstract demonstrates evaluation of the applicability of the Demirjian's method in the studied sample and consequent development of an age predictive equation suitable to the studied sample.
